# Indexing Permafrost Soil Organic Matter Degradation Using High-Resolution Mass Spectrometry

**DOI:** 10.1371/journal.pone.0130557

**Published:** 2015-06-12

**Authors:** Benjamin F. Mann, Hongmei Chen, Elizabeth M. Herndon, Rosalie K. Chu, Nikola Tolic, Evan F. Portier, Taniya Roy Chowdhury, Errol W. Robinson, Stephen J. Callister, Stan D. Wullschleger, David E. Graham, Liyuan Liang, Baohua Gu

**Affiliations:** 1 Environmental Sciences Division, Oak Ridge National Laboratory, Oak Ridge, Tennessee, United States of America; 2 Department of Geology, Kent State University, Kent, Ohio, United States of America; 3 Environmental Molecular Sciences Laboratory, Pacific Northwest National Laboratory, Richland, Washington, United States of America; 4 Biosciences Division, Oak Ridge National Laboratory, Oak Ridge, Tennessee, United States of America; Tennessee State University, UNITED STATES

## Abstract

Microbial degradation of soil organic matter (SOM) is a key process for terrestrial carbon cycling, although the molecular details of these transformations remain unclear. This study reports the application of ultrahigh resolution mass spectrometry to profile the molecular composition of SOM and its degradation during a simulated warming experiment. A soil sample, collected near Barrow, Alaska, USA, was subjected to a 40-day incubation under anoxic conditions and analyzed before and after the incubation to determine changes of SOM composition. A CHO index based on molecular C, H, and O data was utilized to codify SOM components according to their observed degradation potentials. Compounds with a CHO index score between –1 and 0 in a water-soluble fraction (WSF) demonstrated high degradation potential, with a highest shift of CHO index occurred in the N-containing group of compounds, while similar stoichiometries in a base-soluble fraction (BSF) did not. Additionally, compared with the classical H:C vs O:C van Krevelen diagram, CHO index allowed for direct visualization of the distribution of heteroatoms such as N in the identified SOM compounds. We demonstrate that CHO index is useful not only in characterizing arctic SOM at the molecular level but also enabling quantitative description of SOM degradation, thereby facilitating incorporation of the high resolution MS datasets to future mechanistic models of SOM degradation and prediction of greenhouse gas emissions.

## Introduction

A recent estimate shows that the northern permafrost region contains ~1672 Pg of soil organic carbon (C), which accounts for ~ 50% of the estimated global belowground organic C pool [1]. Rising temperatures associated with climate change are expected to increase the availability of this C source for microbial degradation and thus lead to increased production of greenhouse gases (GHG), CO_2_ and CH_4_ [2]. Currently, global climate models that are used to project future climate and C cycle feedbacks conceptualize belowground C as residing in several interconnected pools with varying intrinsic decomposition rates or turnover times [3,4]. However, in these models a large source of uncertainty exists in the chemical composition and distribution of C pools in soil. The physical and chemical differences among these pools are poorly defined [5,6], because soil organic matter (SOM) consists of heterogeneous C sources with varying compositions (ranging from simple sugars, organic acids and amino acids to more complex polymers such as cellulose, lignin, lignocellulose, etc.) and structural characteristics (degrees of polymerization and aromaticity) [5–8]. Furthermore, the distribution of these C pools is highly dynamic with respect to time, location, and their association with minerals; the latter of which affects their recalcitrance to microbial degradation [5,6]. Mechanistic models of C cycling that represent specific soil microbial processes are actively being developed [9–13]; these will require more specific descriptions of SOM composition.

The research community has thus highlighted Arctic SOM chemistry as a critical aspect of C and nutrient cycling [14–19]. These Arctic soils contain high proportions of SOM that is frozen and inaccessible to microbial degradation for most of the year. Unlike other major considerations such as soil moisture and temperature, which can be readily measured in the field, our understanding of SOM composition has remained limited as a result of the vast diversity of SOM and the lack of routine analytical techniques capable of profiling thousands of individual SOM compounds. The inability to characterize SOM has limited our understanding of its chemical and biological interactions, and thus to model and predict its impact on global C cycling and climate [9–13]. Accurate determination of the composition and distribution of C pools, and subsequently the development of meaningful indices for the global C cycling model will thus be a key step toward quantifying and predicting the role of SOM in GHG emissions from thawing permafrost.

We have applied the state-of-the-art Fourier transform ion cyclotron resonance mass spectrometry (FTICR-MS) to probe the composition of SOM and to generate indices that can potentially be used to model the impact of a warming climate on microbial degradation of SOM. FTICR-MS is capable of identifying the molecular formulae of thousands of individual compounds in SOM with minimal sample preparation. Further, SOM analytes are injected into the instrument with electrospray ionization (ESI-FTICR-MS), a “soft ionization” technique that introduces large intact organics to the MS [20], whereas other techniques such as pyrolysis gas chromatography MS modify the native structures prior to mass measurements [21,22]. The number of studies that utilize FTICR-MS has steadily increased in the literature in recent years, including several examples of profiling organic matter derived from Arctic regions [23–26], though the application of FTICR-MS for SOM profiling remains in a nascent stage, and its potential to aid the development of improved process models has not been realized.

FTICR-MS analysis of natural organic matter (NOM) mirrors the fields of metabolomics [27] and proteomics [28,29] as a powerful technique capable of profiling complex mixtures of biologically-derived organic molecules. Thus we refer to the methodology as NOM omics or, simply, "NOMics". Using this approach, here we present the results of a simulated soil warming experiment as proof-of-concept, in which a mineral soil sample was incubated anaerobically at elevated temperature and the SOM components before and after the incubations were extracted and then analyzed with FTICR-MS. We introduce a CHO index that describes the relative H, O, and C content in organic molecules and may thus provide a means to numerically codify components of SOM. Further, the CHO index could be used to incorporate molecular oxidation into computer algorithms that model microbial degradation of SOM. Based on our results, we propose that the NOMics approach may be applied to field and controlled studies of microbial degradation of SOM and help to address the question: How do different SOM compounds degrade in a warming environment?

## Material and Methods

### Sample collection and soil organic matter extraction

A soil core was collected frozen from a low-centered polygon soil at Barrow Environmental Observatory (BEO) in Barrow, Alaska (N 71°16.893', W 156°36.617'), with permission from BEO Authority, and the field study did not involve endangered or protected species. The core was stored at -20°C in a freezer until use, and the mineral horizon soil was then thawed overnight and homogenized in a glove bag under a N_2_/H_2_ atmosphere (methods detailed in Roy Chowdhury et al. [30]). The soil is classified as silt in texture and contains abundant iron oxides with 1M KCl-extractable ferrous Fe(II) up to 0.8 g/kg dry soil [30,31]. A simulated soil warming experiment was performed at room temperature (22°C) to accelerate the degradation process under anoxic conditions so that changes of soil organic carbon compositions could be examined during a 40-day incubation period using FTICR-MS. Samples (15 g wet soil) were kept in 60-mL serum bottles fitted with rubber butyl septa. Following the incubation, SOM compounds were extracted into operationally defined fractions based on their solubility in water (0.01M KCl), acid (0.1M HCl), and base (0.1M NaOH) [32]. The base extraction was carried out under N_2_ atmosphere to minimize the oxidation of SOM, and the extract was neutralized immediately with dilute HCl to pH ~7, in which SOM remained soluble [33]. We denote the water-soluble fractions of SOM extracted at day 0 (before incubation) and day 40 (after incubation) as WSF0 and WSF40, respectively, and likewise the base-soluble fractions as BSF0 and BSF40, and so on. Total dissolved organic carbon (DOC) concentrations in the extracts were analyzed using a Shimadzu Total Organic Carbon (TOC) Analyzer. Total SOM before incubation was determined by the combustion technique [30] and was 64.2±11.4 mg C/g dry soil. The extraction procedures recovered 39.5±10.3 mg C/g dry soil (N = 3), and thus the recovery was 61.5%, with a relative distribution of 3.4±0.2%, 3.1±0.2%, and 93.5±0.1% of all solubilized SOM in the WSF, ASF, and BSF, respectively. However, the acid extracts were not analyzed by FTICR-MS due to a high iron content resulting in the precipitation of a large portion of the organic matter when neutralized; only water and base-soluble samples at day 0 and day 40, were analyzed by FTICR-MS as described below.

### FTICR-MS analysis of SOM extracts

To obtain elemental compositions for extracted SOM samples, analysis was performed using a 15T FTICR-MS (Bruker SolariX, Billerica, MA) outfitted with standard ESI interface. The mass spectrometer was set to acquire data in positive mode due to the use of KCl and/or HCl in SOM extraction. Previous studies showed that NOM-chloride adducts usually exhibit higher mass defects (> 0.6) in the negative mode [34], but they appeared as minor peaks in our positive mass spectra (S1 Fig). There were alkali metal adducts, mainly K^+^ and Na^+^, observed along with protonated H^+^ ions, which are common in positive mode FTICR-MS analyses [35,36]. However, unlike the Cl^-^ adducts that would dramatically suppress the signal of organic molecules under negative mode FTICR-MS, the K^+^ or Na^+^ adducts have similar ionization efficiency as protonated ions under positive mode (S1 Fig). All samples were diluted with methanol (1:1, v:v) to a concentration of 20 mg C/L and directly infused using a Hamilton syringe at a flow rate of 2 μL/min. The coated glass capillary temperature was set to 180°C and the electrospray voltages were optimized for each sample. The ESI signal was allowed to stabilize for 5 min, ion accumulation time was 0.1 sec for 96 scan averages co-added, time of flight was set to 0.65 ms, and Q1 was set to 150 *m/z*. The instrument was externally calibrated prior to sample analysis [37], and the syringes and tubes were flushed with 50/50 methanol/water (v/v) between samples.

### Molecular assignments to FTICR-MS measurements

Mass spectra contained predominantly singly charged compounds as indicated by the one-Thomson difference (Δm/z = 1.00335) between monoisotopic peaks and higher isotope peaks containing one ^13^C atom (S1 Fig), as observed in previous studies [37–39]. Spectra were internally calibrated using series of organic acids present in the soil extracts [37]. Molecular formulae assignments were made using a modified version of the Compound Identification Algorithm (CIA) described by Kujawinski and coworkers [20] and updated as described subsequently [38]. Formulae were assigned for peaks with a signal to noise (S/N) ratio > 7 and *m/z* range between 200 and 1200. A mass error window of 1.0 ppm was used to compute the possible molecular formulae to MS peaks less than 500 *m/z*. The elements C, H, O, N, S, P, Na, and K were used to generate putative assignments of elemental compositions for individual molecules in SOM. The formula with the lowest number of non-oxygen heteroatoms (N + S + P) was then assigned [20]. In the second step of CIA, *m/z* values above 500 were assigned by means of an extension algorithm that added common CH_2_ building block to the *m/z* of formulae assigned to smaller (< 500 *m/z*) compounds. When the *m/z* of a CH_2_ group and already assigned compound summed to an *m/z* that was observed above 500, the formula assigned to the smaller compound was then appended by the atoms of CH_2_ and the new formula was assigned to the larger compound. To remove likely false positives, lists of assigned formula were filtered and restricted according to the following criteria: H/C > 0.3, H ≤ 2C + N + S + 2P + 2, N/C ≤ 0.5 per the recommendation of Koch et al. [40]. We observed < 20 organic molecules in each sample, where the protonated H^+^ and metal ion (K^+^ or Na^+^) adduct peaks were both shown in a mass spectrum (S1 Fig). Subsequently, the peak with higher intensity was selected as the corresponding molecule. For example, for WSF0 sample, a neutral molecule C_22_H_32_O_9_ was identified by the presence of two ion peaks: protonated ion C_22_H_33_O_9_^+^ (measured m/z = 441.211599, and intensity = 1016734) and K^+^ adduct ion C_22_H_32_O_9_K^+^ (measured m/z = 479.168219, and intensity = 3822028). The high intensity peak (m/z 479.168219) was thus selected to represent the molecule C_22_H_32_O_9_, and its intensity used for magnitude-weighted calculations (Table 1).

**Table 1:** Number weighted (Mean _#_) and magnitude-weighted mean (Mean _w_) properties for SOM extracts from a simulated soil warming experiment. The CHO index was calculated as (2×[*O*]–[*H*])/[*C*].

	Water soluble fraction (WSF)				Base soluble fraction (BSF)			
	Day 0		Day 40		Day 0		Day 40	
	Mean _#_	Mean _w_	Mean _#_	Mean _w_	Mean _#_	Mean _w_	Mean _#_	Mean _w_
***CHO formulae***								
Formulae	C_26.4_H_37.1_O_10.8_	C_26.8_H_36.2_O_12.3_	C_27.4_H_38.9_O_10.4_	C_28.5_H_37.0_O_11.5_	C_29.7_H_39.0_O_6.9_	C_28.8_H_39.4_O_7.3_	C_32.1_H_43.3_O_7.0_	C_31.4_H_44.0_O_7.3_
O:C	0.45	0.53	0.42	0.48	0.27	0.28	0.26	0.28
H:C	1.45	1.44	1.46	1.39	1.30	1.37	1.32	1.39
CHO Index	-0.55	-0.38	-0.62	-0.44	-0.76	-0.80	-0.80	-0.84
***CHON formulae***								
Formulae	C_32.3_H_44.2_O_8.6_N_2.8_	C_35.1_H_46.9_O_8.2_N_3.0_	C_35.6_H_51.6_O_8.0_N_2.6_	C_31.6_H_46.2_O_5.0_N_3.4_	C_35.9_H_53.1_O_6.0_N_2.8_	C_31.5_H_46.1_O_5.3_N_3.4_	C_35.7_H_52.3_O_5.8_N_2.9_	
O:C	0.32	0.27	0.26	0.23	0.19	0.19	0.20	0.19
H:C	1.36	1.34	1.45	1.41	1.44	1.49	1.43	1.47
N:C	0.10	0.09	0.08	0.08	0.13	0.10	0.13	0.10
CHO Index	-0.71	-0.80	-0.93	-0.95	-1.06	-1.12	-1.04	-1.10

## Results

### CHO index and SOM degradation potential

**Fig 1:**
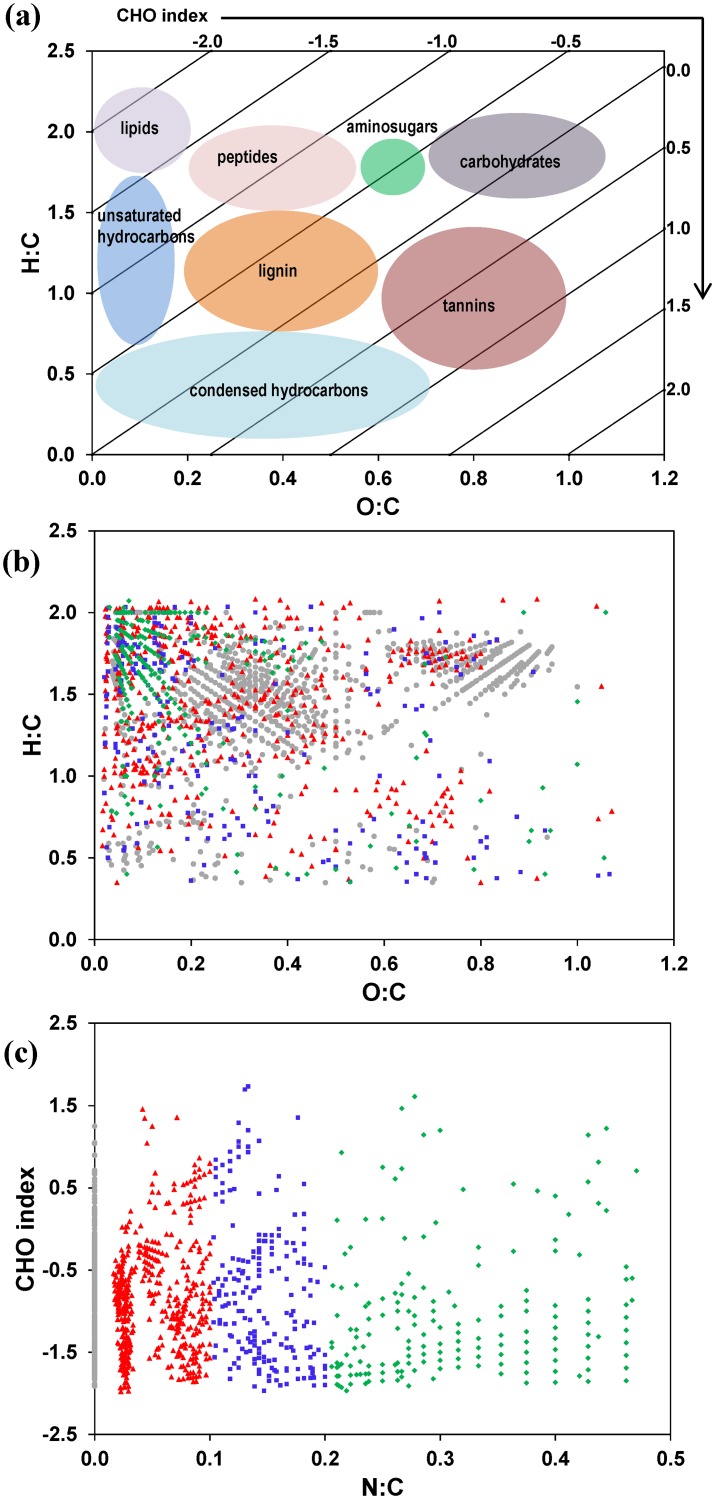
van Krevelen diagram of the compound classes associated with the H:C and O:C-coordinate plane and CHO Index plot with N:C coordinate. **(a)** The CHO index, $\frac{2\times \left[O\right]-\left[H\right]}{\left[C\right]}$, is plotted along the top and right edges of the van Krevelen diagram to illustrate their relationships and associated compound classes of organic matter assigned by Kim et al. [42]. **(b)** Illustration of a van Krevelen diagram for a water-soluble SOM sample (WSF0) extracted from an Arctic soil. Colored symbols represent different molecular components plotted in **(c)**, which is a CHO index plot with molecular components binned according to N:C ratio: N:C = 0 (gray circles), 0 < N:C ≤ 0.1 (red triangles), 0.1 < N:C ≤ 0.2 (blue squares), 0.2 < N:C (green diamonds); Compounds with similar N:C are localized to distinct regions of the van Krevelen diagram in (b).

With a NOMics approach, the numbers of C, H, and O atoms were measured experimentally and presented as the van Krevelen diagram (illustrated in Fig 1a), which plots H:C vs. O:C [41]. While van Krevelen initially utilized these plots to compare bulk elemental ratios (H:C and O:C) in different coal samples, they were first used in NOMics literature to describe the individual components of organic matter identified with FTICR-MS measurements by Kim et al. [42]. Regions of the van Krevelen diagram have been assigned to biomolecular classes of compounds [42]. Although these descriptions are considered illustrative, here we employed a van Krevelen diagram to display different classes of SOM compounds (Fig 1a) along with the CHO index plotted on the top and right edges of the diagram to demonstrate their relationships (described further below). We used WSF0 sample to display the distribution of assigned molecular formulae according to H:C and O:C coordinates (Fig 1b); different color-coded symbols represent different classes of N-containing compounds (Fig 1c), as discussed in detail below. Dense clustering in the middle of the plot (Fig 1b) indicates the prevalence of lignin-like compounds. The O:C < 0.2 formulae are traditionally described as hydrocarbons of varying degrees of saturation, with those near H:C = 2 being predominantly lipid-like. These classifications are reasonable generalizations for structures containing C, H, and O, and also provide a means to directly visualize NOMics datasets and discern qualitative information about different SOM pools. However, the van Krevelen diagram cannot adequately describe components of SOM containing heteroatoms such as N and, more importantly, the quantitative relationships between these C pools (or the O:C and H:C coordinates) and SOM degradation potential cannot be established.

Currently there are two indices (i.e., double-bond equivalents, DBE) [43] and aromaticity [44] commonly used to characterize NOM compositions from FTICR-MS, but they are not directly related to the oxidation state and degradation potential of NOM. Using the concept of the oxidation state of organic C [45], here we propose to use a CHO index: the O:H ratio normalized to C (Eq 1), to describe SOM degradation potential. The CHO index plots each SOM molecule along a spectrum from highly reduced (high relative H content) to highly oxidized (high relative O content) (Fig 1a):

$$CHO\;Index=\frac{2\times \left[O\right]-\left[H\right]}{\left[C\right]},$$ (1)

where [*O*], [*H*], and [*C*] are the number of hydrogen, oxygen and carbon atoms in a molecule, respectively. The oxidation state of C can vary between -4 (e.g., 4 C-H bonds as in CH_4_), and +4 (e.g., 2 C = O bonds as in CO_2_). Likewise, possible CHO index values for organic carbon molecules containing only C, H, and O can fall between -4 and 4, with CH_4_ and CO_2_ representing the low and high ends, respectively. Note that CHO index = 0 when the number of hydrogen atoms is twice the number of oxygen atoms, as is the case in a carbohydrate such as glucose and glucose polymers, e.g., starch and cellulose. Higher CHO index values can be attributed to more oxidized compounds, e.g., tannic acid (C_76_H_52_O_46_, CHO index = 0.53), while lower values denote reduced molecules, e.g., oleic acid (C_18_H_34_O_2_, CHO index = –1.67). As an example, common monolignols, e.g., *p*-coumaryl alcohol, coniferyl alcohol, and sinapyl alcohol, which constitute the bulk of lignin polymers, typically have CHO index scores below -0.5 (-0.60, -0.55, and -0.67, respectively, for those named as examples).

By condensing C, H, and O information into a single value, CHO index can also be plotted against heteroatom information such as N, while both H and O remain represented. This is a significant advantage of using CHO index so that the distribution of N in the identified SOM compounds (Fig 1b) can be directly visualized by plotting CHO index vs. N:C (Fig 1c). Modifications to the van Krevelen diagram (H:C plotted against O:C) are made by replacing one of the axes with a different elemental ratio, e.g., N:C, S:C, P:C, in order to visualize the distribution of heteroatoms within SOM mixtures [41,42]. By using CHO index, both H and O remain represented, and the distribution of heteroatom such as nitrogen in the identified compounds is demonstrated (Fig 1c). The organization of data in Fig 1c, particularly between N:C = 0.1 and N:C = 0.2, indicated the presence of several series of structurally related compounds. This observation was highlighted by applying the color code, denoting N:C ≤ 0.1 (red triangles), 0.1 < N:C < 0.2 (blue squares), and N:C ≥ 0.2 (green diamonds), in both the van Krevelen diagram (Fig 1b) and the CHO index plot (Fig 1c). Structures with 0 < N:C ≤ 0.1 (red triangles) were mostly observed above H:C = 1.0, with a high concentration of coordinates overlapping the region of the plot associated with proteins. Compounds with N:C ≥ 0.2 (green diamonds) were found primarily above H:C = 1.5, the region traditionally assigned to lipids (see Fig 1a), while those with 0.1 < N:C ˂ 0.2 (blue squares) were generally present at H:C < 1.5. These measurements may be indicative of compounds containing a high number of amino groups (blue squares and green diamonds); the green diamonds may include SOM compounds with aliphatic and/or primary amines, while the blue squares denote SOM that may have incorporated secondary or even tertiary amines as well as N-heterocycles [46].

### Relating CHO index to SOM transformation in a soil warming experiment

**Fig 2:**
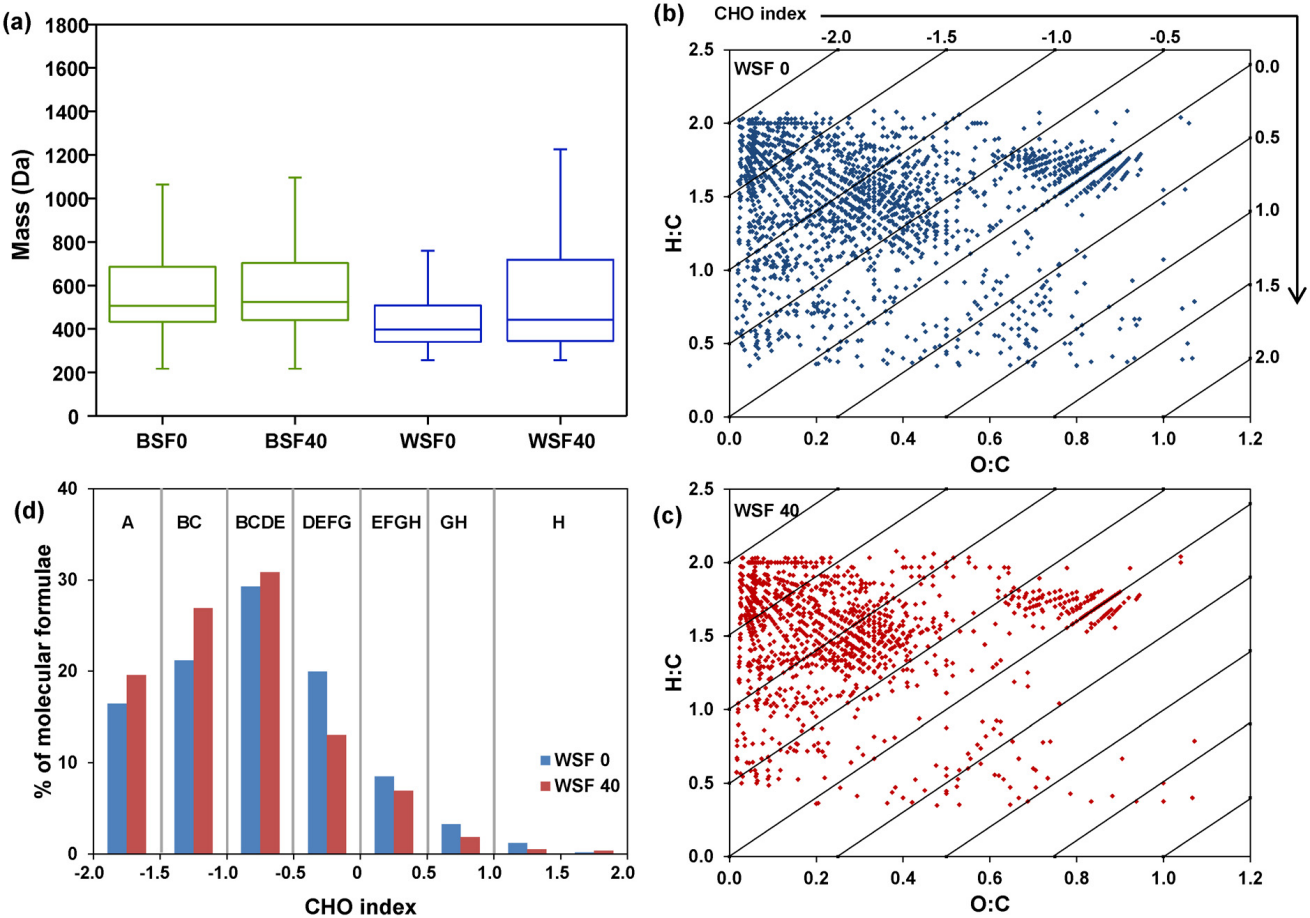
Molecular distribution of extracted SOM compounds from a 40-day soil warming incubation experiment. (a) Box-and-whisker plots of the mass distribution of SOM compounds, including the base-soluble fraction (BSF) at day 0 (BSF0) and day 40 (BSF40) and the water-soluble fraction (WSF) at day 0 (WSF0) and day 40 (WSF40). **(b and c)** van Krevelen diagram along with CHO index showing the molecular distribution of WSF SOM compounds before (b) and after (c) incubation. **(d)** Percentages of molecular formulae identified with CHO index values between -2 and 2 before and after soil incubation and are normalized to the total number of formulae displayed in (b) and (c). Compound classes are labeled above colored bars as follows: (A) lipids, (B) unsaturated hydrocarbons, (C) peptides, (D) aminosugars, (E) carbohydrates, (F) lignin, (G) condensed hydrocarbons, (H) tannins.

The extraction procedures described above recovered 39.5±10.3 mg C/g dry soil, in which 3.4±0.2% and 93.5±0.1% of the solubilized SOC were present in the WSF and BSF, respectively. The amount of total C degraded (measured by CO_2_ and CH_4_ production) during the entire incubation period (40 days) was less than 0.25 mg C/g dry soil (or < 1%) (S2 Fig). Box-and-whisker plots (Fig 2a) illustrate the statistical distribution of the mass values that were assigned molecular formulae in the BSF0 and WSF0 (before incubation) and BSF40 and WSF40 (after incubation). Many of the lower molecular weight compounds observed in WSF0 were not present in WSF40, leading to the widening of the interquartile range observed in the WSF40 sample. Thus, the water-soluble carbon was apparently transformed over the course of the incubation. However, the mass distribution of compounds in the BSF changed little over the same incubation period.

We subsequently calculated and compared the average molecular formulae, CHO Index, and the number-averaged and magnitude-weighted elemental ratios of O;C, H:C, and N:C in WSF0, WSF40, BSF0, and BSF40 using previously established methods [38,39]. We note that the elemental compositions calculated from the identified molecular formulae are limited to those ionizable and detectable by FTICR-MS since FTICR-MS is not quantitative due to its selective ionization efficiencies with different compounds [38,39]. However, when comparing samples under identical conditions (e.g., WSF0 vs. WSF40), these compositions are useful to provide insights into the differences and/or changes of SOM samples. We performed calculations by separating two major groups of formulae: CHO and CHON, and the results are listed in Table 1. Evidently, major changes occurred between WSF0 and WSF40, particularly within the CHON formulae group, in which the number-averaged and magnitude-weighted CHO index decreased from -0.71 to -0.93 and from -0.8 to -0.95, respectively. These changes in CHO index are much greater than those of elemental ratios (i.e., O:C, H:C, and N:C). However, little changes occurred between BSF0 and BSF40, confirming that the WSF SOM is more labile than the BSF SOM. These results demonstrate the advantages of using CHO index, which is more sensitive than the elemental ratios used in van Krevelen diagram to indicate the degradation potential of SOM.

**Fig 3:**
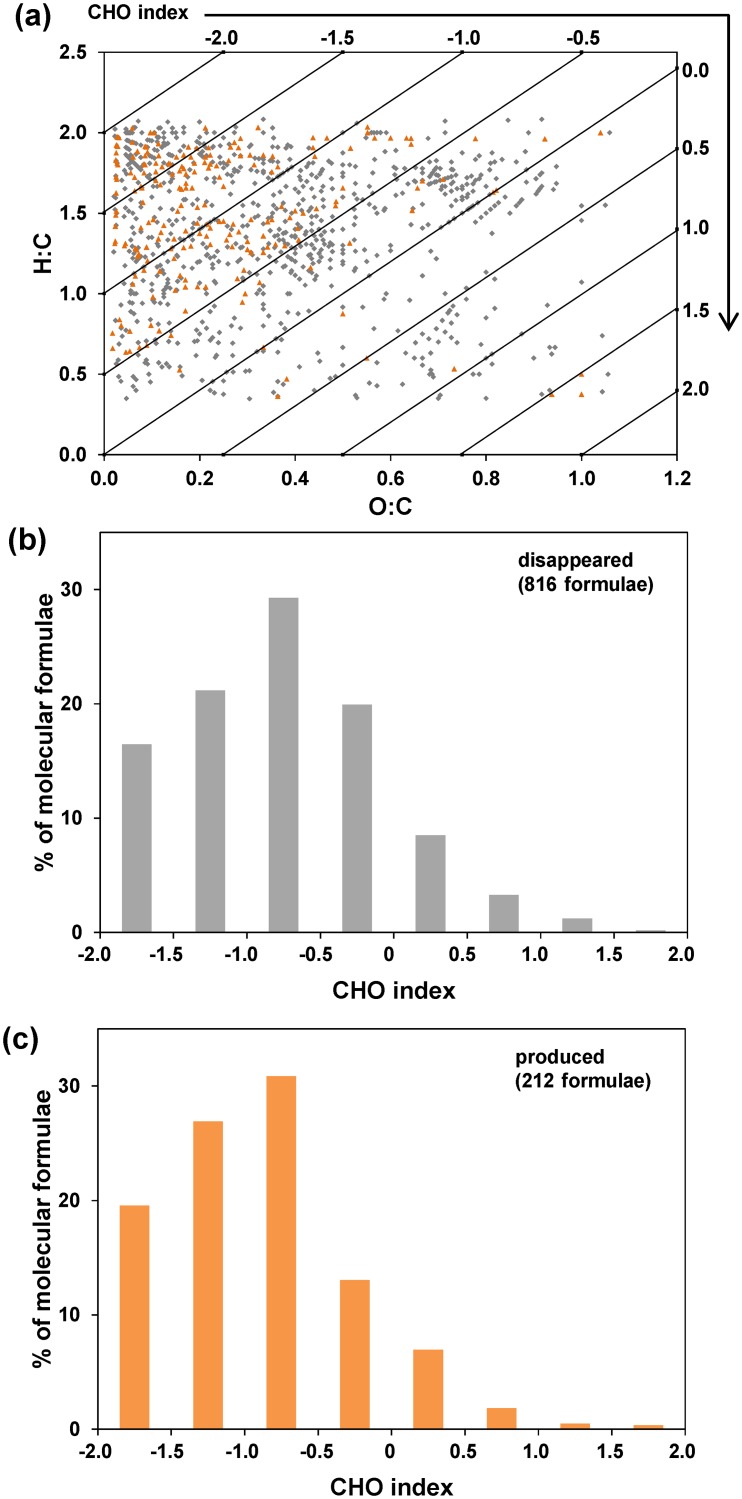
Illustration of the molecular formulae that were either degraded (grey color) or produced (orange color) in the WSF SOM during the 40-day incubation experiment. **(a)** van Krevelen diagram along with CHO index showing the molecular formulae that were either degraded (grey color) or produced (orange color) in the WSF SOM after incubation. **(b)** CHO Index plots of the number of molecular formulae that were either disappeared in the WSF after incubation (normalized to the total of 816 formulae) or **(c)** produced after incubation (normalized to the total of 212 formulae).

We further compared the CHO index and the van Krevelen diagram using all molecular formulae identified in WSF0 and WSF40 samples (Fig 2b and 2c). Data in BSF0 and BSF40 were not plotted because of small changes observed (as described above). The van Krevelen plots showed that WSF40 sample contained lower numbers of molecular formulae at lower H:C and higher O:C ratios than those of WSF0. These changes, however, are difficult to discern quantitatively in the van Krevelen plot but can be readily discerned in the CHO index plot (Fig 2d). The number of molecular assignments that had specific CHO index values between -2 and 2 was plotted for comparing WSF0 and WSF40 SOM fractions, where the major biomolecular classes represented at each CHO index score are shown. Following incubation, we clearly observed an increased relative abundance of molecules with CHO index < –0.5 but a decreased relative abundance of molecules with CHO Index > –0.5 over the 40-day incubation period (Fig 2d). This is further illustrated by comparing molecules that were either disappeared or produced during the 40-day incubation period (Fig 3). We observed 816 molecular formulae with CHO index mainly between -1.0 and 0 disappeared (Fig 3b), whereas about 212 molecules mostly with lower CHO index between -2.0 and -0.5 produced (Fig 3c). Clearly a large number of SOM molecules that were degraded falls in compound classes of aminosugars and carbohydrates (~ 55%, CHO index between -1.0 and 0), followed by peptides and unsaturated hydrocarbons (~ 30%, CHO index between -2.0 and -1.0) (Fig 3b and 3c). Some of these molecules were transformed to form new molecules with CHO index shifted to lower values (< –1.0) (Fig 3c). These results thus indicate that the CHO index is a useful proxy for the degradation potential of different pools of SOM and may be used to improve mechanistic models of SOM degradation [12,13].

### Relating CHO index to molecular size and heteroatom incorporation

**Fig 4:**
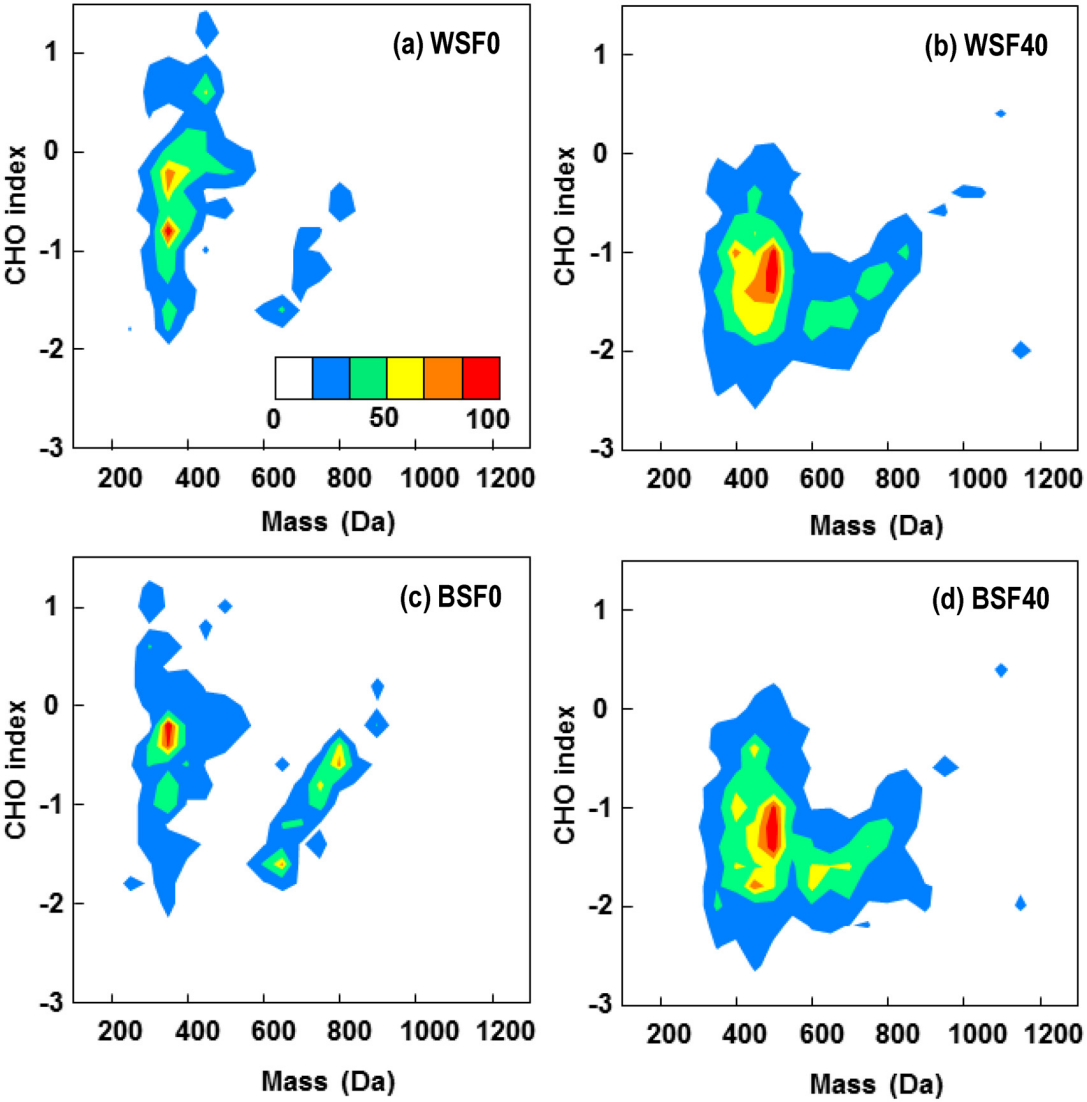
Heatmaps for CHO index as a function of molecular mass of extracted SOM compounds before and after the soil warming experiment. The color bar represents the relative abundance of compounds identified in each of the SOM extract: **(a)** WSF0, **(b)** WSF40, **(c)** BSF0, and **(d)** BSF40. A positive correlation between CHO index and mass can be observed for mass > 600 Da.

The molecular size of SOM compounds also has been previously linked to microbial degradation potential [47,48]. Heatmaps of the CHO index and mass depict how the CHO index values in the WSF0 (Fig 4a) are skewed toward the positive with CHO index above -1, while most molecular masses are between 250 and 600 Da. The decreased relative abundance of low mass compounds in WSF40 (Fig 4b) coincided with an increase in the relative abundance of compounds larger than 600 Da, which is consistent with increased numbers of compounds with CHO index values between -0.5 and -2.0 (Fig 3c) and may be attributed to the more recalcitrant SOM components. BSF (Fig 4c and 4d), on the other hand, was characterized by a majority of compounds concentrated at the negative end of the CHO spectrum. Interestingly, a clear positive trend between mass and CHO index was observed from *c*.600 to 1200 Da in both BSF0 and BSF40 samples (S3 Fig). By comparing mass to H:C and O:C ratios, we observed that H:C decreased while O:C increased with increasing molecular mass from *c*.600 to 1200 Da, suggesting both properties contribute to the observed trend in CHO index.

**Fig 5:**
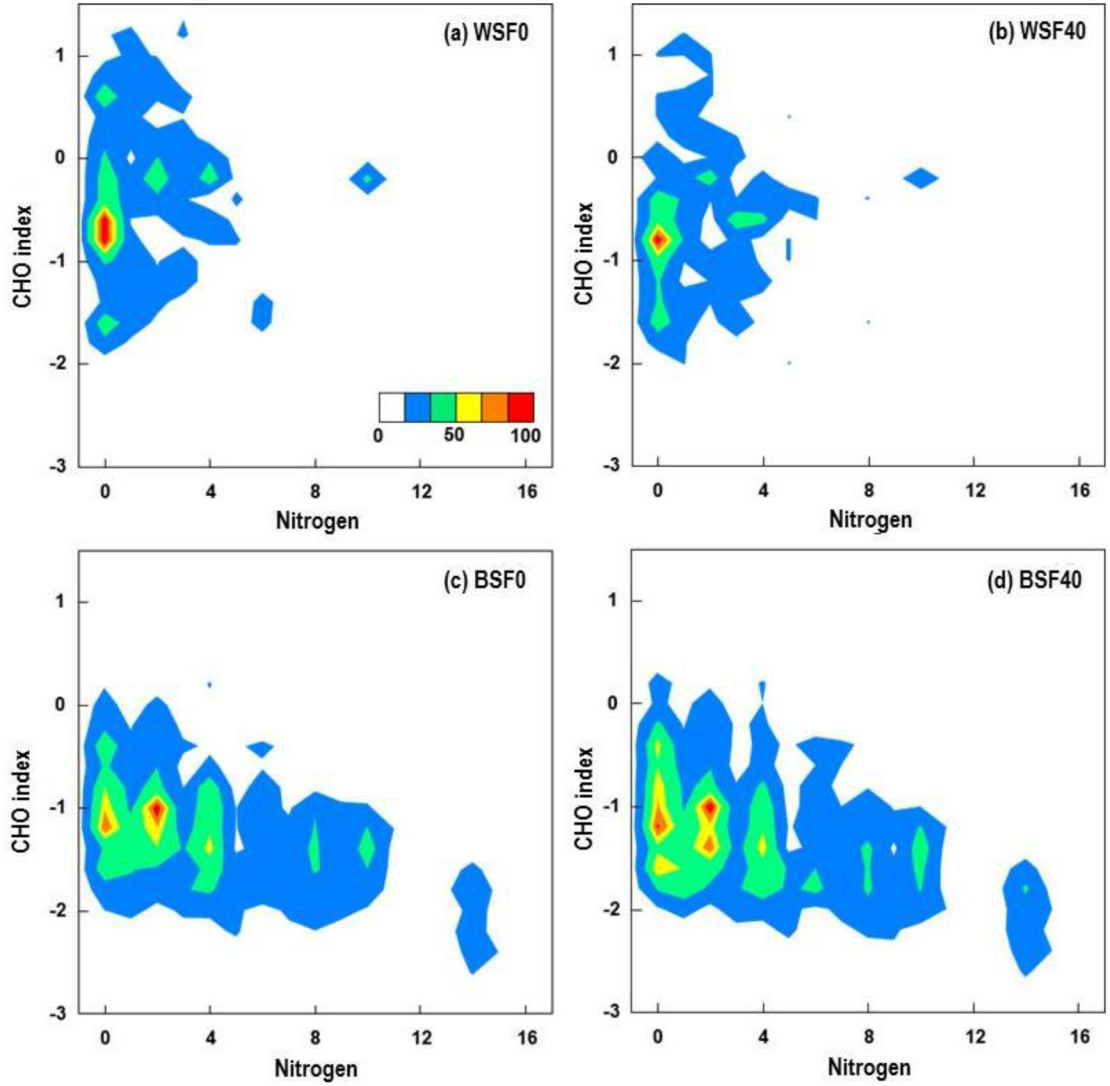
Heatmaps for CHO index as a function of the number of N atoms in extracted SOM compounds identified in (a) WSF0, (b) WSF40, (c) BSF0, and (d) BSF40. The color bar represents the relative abundance of compounds identified in each of the extract. Note the “island” formations observed for even numbers of N atoms in the BSF samples.

CHO index was also plotted versus the number of N atoms to visualize trends in CHO index as a function of N (Fig 5). The WSF0 and WSF40 samples contained SOM molecules predominantly two or fewer nitrogen atoms, with 0 the most frequent number in a molecular composition (Fig 5a and 5b). By contrast, the BSF in Fig 5c and 5d, showed a regular pattern of N incorporation, with “islands” observed at even numbers of N atoms (e.g., at 0, 2, 4, 8, etc.). This intriguing observation was confirmed by analyzing three separate mineral soil BSF samples from the same soil core; the pattern of N atoms was observed in each. The negative correlation between CHO index and N may indicate that N could have been a component of amine functional groups or N-heterocycles [46].

## Discussion

This study utilized a NOMics approach to provide a molecular-level characterization of SOM compounds in an Arctic soil warming experiment. A numerical CHO index based on molecular carbon, hydrogen, and oxygen data was utilized to codify SOM components according to their relative C, H, and O content. We observed that low molecular weight, water-extractable SOM compounds (WSF) with CHO index scores at 0 to -1.0 were the most readily degraded under warming (Fig 3). However, compounds in the BSF SOM did not change appreciably over the same warming period, suggesting that 40 days at elevated temperature were insufficient to observe degradation of this more stabilized pool of SOM. The WSF number-weighted mean CHO index shifted from -0.55 to -0.62 over the 40-day incubation period in the CHO only formulae group, but it shifted from -0.71 to -0.93 in the CHON formulae group (Table 1), indicating that the N-containing compounds are among the most vulnerable to microbial degradation. The CHO index is also shown to be more sensitive than elemental ratios (i.e., O:C and H:C in van Krevelen diagram) in describing changes of molecular composition or degradation of SOM.

Positive correlations were observed between CHO index and mass in both WSF and BSF samples above 600 Da and may be attributed to lignins and other aromatic compounds stabilized through mineral interactions, particularly iron oxyhydroxides which are abundant in these soils [30,31]. However, we note that the low end of the mass range that was analyzed by FTICR-MS measurements was 200 *m/z* so that the production and consumption of low-molecular weight organic compounds such as small organic acids and alcohols are not addressed by the FTICR-MS measurement approach. Alternative analytical strategies including high-performance liquid chromatography or ion chromatography are needed to monitor the important role these compounds play in GHG production. We also observed an apparently ordered pattern of nitrogen incorporation into the arctic SOM. Based on the established body of literature and our measurements, we speculate that a fraction of nitrogenous SOM is immobilized through interactions with soil mineral surfaces. Strong immobilization of organic N has been described previously in a study of fertilization in arctic soils [49] and similarly in an investigation of the effects of freeze-thaw events in alpine tundra [50]. Electrostatic, physicochemical, and hydrophobic forces (based on H:C and C:O ratios) may all have contributed to the stabilization of this SOM pool against microbial degradation and extraction with water. It has been postulated that as much as 35% of organic-N could be distributed as N-heterocycles in SOM [46], although direct measurements by techniques such as ^15^N-nuclear magnetic resonance spectroscopy have not been able to detect any specific compounds due to the heterogeneous nature of these SOM pools, as observed with FTICR-MS analysis herein.

We therefore demonstrate that CHO index is useful not only in describing arctic SOM at the molecular level but also directly visualizing the distribution of heteroatoms such as N in the identified SOM compounds. As a single numerical parameter, the CHO index allows to quantitatively describe the degradation potential of different classes of SOM components, which is significant advantage over the van Krevelen diagram, thereby facilitating the incorporation of the high resolution MS datasets to future models of SOM degradation. Current models assume a number of discrete SOM pools with varying degradation potentials [3,4]. Therefore accurate determination of the composition, distribution, and degradation potential of C pools is an important step toward developing meaningful indices, which could then be used for predicting global C cycling and the role of SOM in GHG emissions from thawing permafrost. The NOMics approach is efficient for generating detailed SOM datasets, although a current challenge is the limited access to the instrumentation by the science community and the need for high-throughput data analysis strategies. By performing larger scale studies of this kind in the future, it can be exploited to provide insights into the molecular details of microbial degradation of SOM, including how it may transform in a warmer climate.

## Supporting Information

S1 Fig Positive mode ESI-FTICR mass spectra of **(a)** the water-soluble fraction (WSF) and **(b)** the base-soluble fraction (BSF) SOM before and after soil incubation (see text for additional details).
(PDF)

S2 Fig Cumulative gas production (carbon dioxide (CO_2_) and methane (CH_4_)) during a simulated 40-day warming incubation experiment with an Arctic mineral soil obtained from Barrow, Alaska, USA. (PDF)

S3 Fig Plots of O:C vs. mass and H:C vs. mass for BSF0 (a,b) and BSF40 (c,d) SOM samples. (PDF)
