# Supplementary material for: Indexing Permafrost Soil Organic Matter Degradation Using High-Resolution Mass Spectrometry
Source: PLoS One. 2015 Jun 12;10(6):e0130557. doi: 10.1371/journal.pone.0130557 (PMC4467038; doi:10.1371/journal.pone.0130557)
Supplement: S1 Fig — Positive mode ESI-FTICR mass spectra of (a) the water-soluble fraction (WSF) and (b) the base-soluble fraction (BSF) SOM before and after soil incubation (see text for additional details). (PDF) [file pone.0130557.s001.pdf]

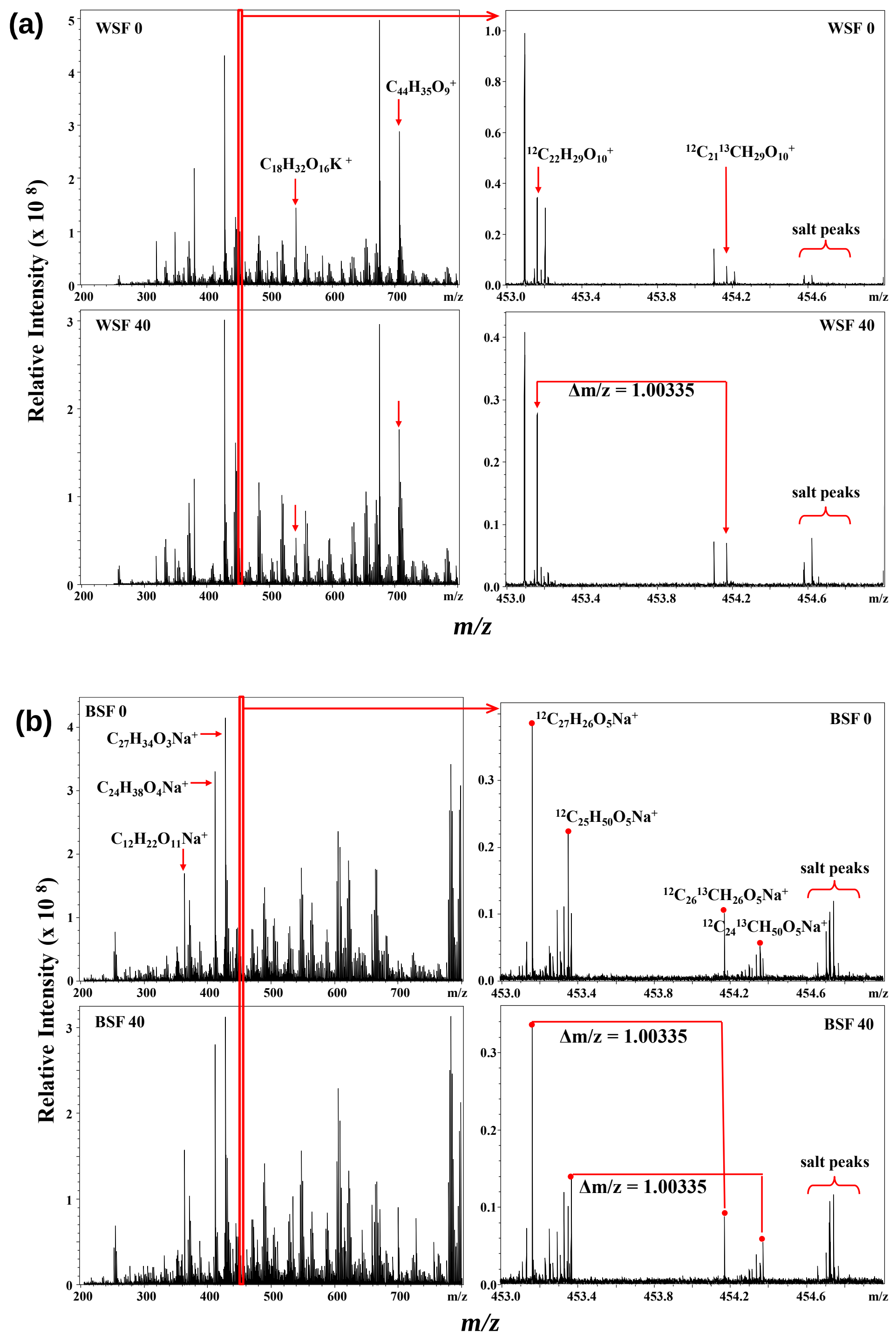

Figure S1. Positive mode ESI-FTICR mass spectra of **(a)** the water-soluble fraction (WSF) and **(b)** the base-soluble fraction (BSF) SOM before (WSF0, BSF0) and after (WSF40, BSF40) incubation (see text for additional details). Left figures show the broad band spectra (m/z 200-800), and Right panel is the expanded region (m/z 453.0-455.0) of the left panel. Several CHO formulae peaks are denoted. The corresponding ^{13}C isotopic peaks were observed indicating singly-charged situation. Minor peaks were observed with mass defect > 0.6 and could be attributed to salt peaks from DOM-Chloride adducts.
