# Supplementary material for: Indexing Permafrost Soil Organic Matter Degradation Using High-Resolution Mass Spectrometry
Source: PLoS One. 2015 Jun 12;10(6):e0130557. doi: 10.1371/journal.pone.0130557 (PMC4467038; doi:10.1371/journal.pone.0130557)
Supplement: S2 Fig — (PDF) [file pone.0130557.s002.pdf]

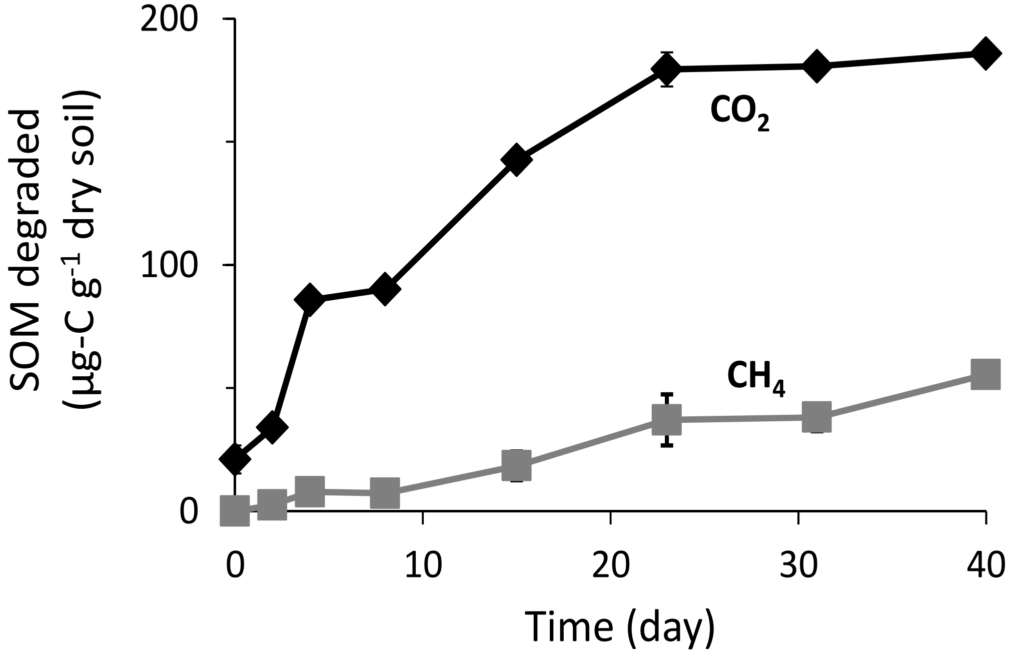

Figure S2. Cumulative gas production (carbon dioxide (CO_2) and methane (CH_4)) during a simulated 40-day warming incubation experiment with an Arctic mineral soil obtained from Barrow, Alaska, USA.
