# Supplementary material for: Indexing Permafrost Soil Organic Matter Degradation Using High-Resolution Mass Spectrometry
Source: PLoS One. 2015 Jun 12;10(6):e0130557. doi: 10.1371/journal.pone.0130557 (PMC4467038; doi:10.1371/journal.pone.0130557)
Supplement: S3 Fig — (PDF) [file pone.0130557.s003.pdf]

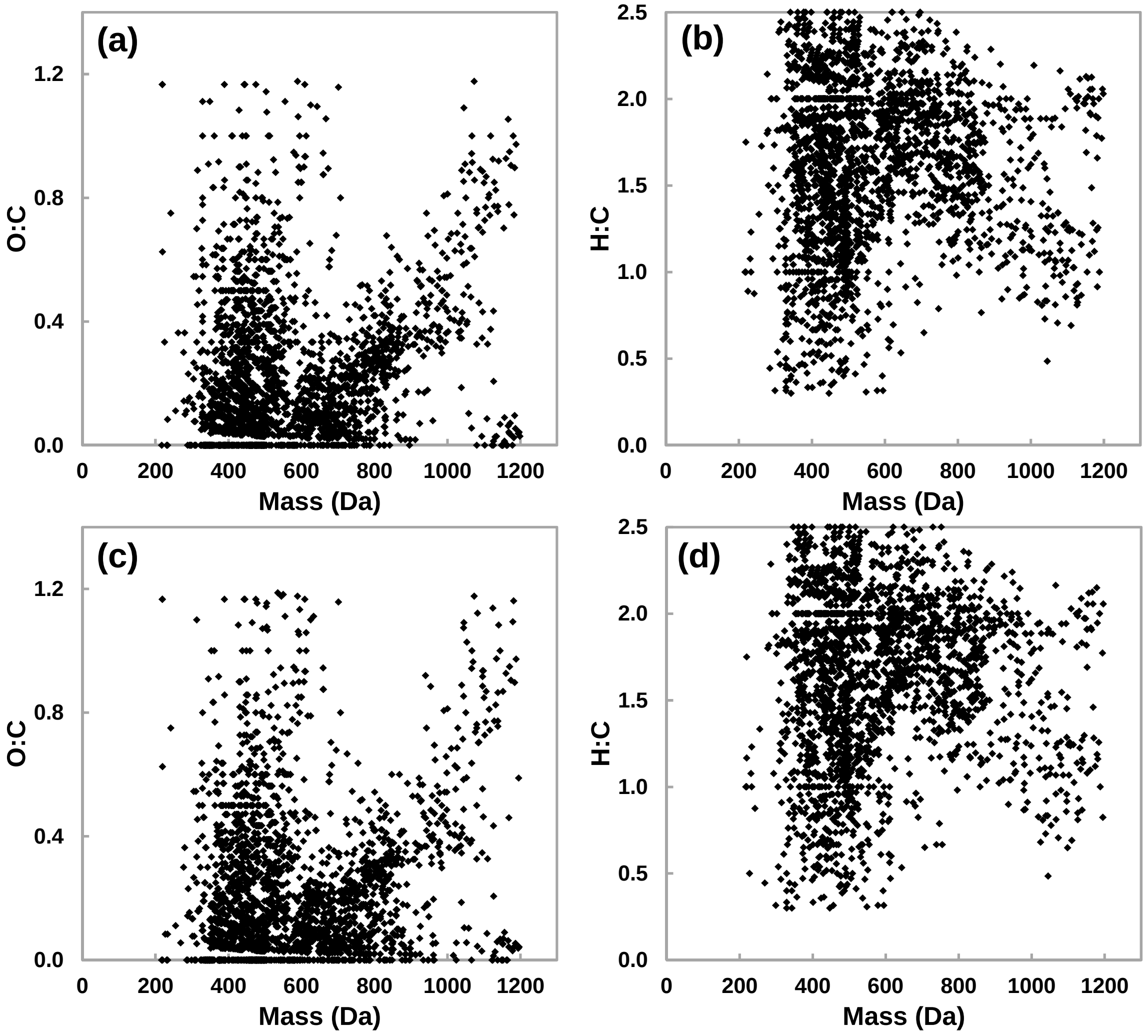

Figure S3. Plots of O:C vs. mass and H:C vs. mass for BSF0 (a,b) and BSF40 (c,d) SOM samples. Distributions of O:C and H:C as functions of mass for the BSF reveal a positive correlation between O:C and mass and a simultaneous negative correlation between H:C and mass above *m/z* 600 in the BSF.
